# Mechanism of RhoA regulating benign prostatic hyperplasia: RhoA-ROCK-β-catenin signaling axis and static & dynamic dual roles

**DOI:** 10.1186/s10020-023-00734-2

**Published:** 2023-10-20

**Authors:** Shidong Shan, Min Su, Yan Li, Zhen Wang, Daoquan Liu, Yongying Zhou, Xun Fu, Shu Yang, Junchao Zhang, Jizhang Qiu, Huan Liu, Guang Zeng, Ping Chen, Xinghuan Wang, Michael E. DiSanto, Yuming Guo, Xinhua Zhang

**Affiliations:** 1 Department of Urology, Zhongnan Hospital of Wuhan University, 169 Donghu Road, Wuhan, 430071 People’s Republic of China; 2 Department of Gynecological Oncology, Zhongnan Hospital of Wuhan University, Wuhan, China; 3 Department of Surgery and Biomedical Sciences, Cooper Medical School of Rowan University, Camden, NJ USA

**Keywords:** Benign prostatic hyperplasia, RhoA-ROCK, β-catenin, Contraction, Cell growth

## Abstract

**Background:**

The pathogenesis of benign prostatic hyperplasia (BPH) has not been fully elucidated. Ras homology family member A (RhoA) plays an important role in regulating cell cytoskeleton, growth and fibrosis. The role of RhoA in BPH remains unclear.

**Methods:**

This study aimed to clarify the expression, functional activity and mechanism of RhoA in BPH. Human prostate tissues, human prostate cell lines, BPH rat model were used. Cell models of RhoA knockdown and overexpression were generated. Immunofluorescence staining, quantitative real time PCR (qRT-PCR), Western blotting, cell counting kit-8 (CCK-8), flow cytometry, phalloidine staining, organ bath study, gel contraction assay, protein stability analysis, isolation and extraction of nuclear protein and cytoplasmic protein were performed.

**Results:**

In this study we found that RhoA was localized in prostate stroma and epithelial compartments and was up-regulated in both BPH patients and BPH rats. Functionally, RhoA knockdown induced cell apoptosis and inhibited cell proliferation, fibrosis, epithelial-mesenchymal transformation (EMT) and contraction. Consistently, overexpression of RhoA reversed all aforementioned processes. More importantly, we found that β-catenin and the downstream of Wnt/β-catenin signaling, including C-MYC, Survivin and Snail were up-regulated in BPH rats. Downregulation of RhoA significantly reduced the expression of these proteins. Rho kinase inhibitor Y-27632 also down-regulated β-catenin protein in a concentration-dependent manner. However, overexpression of β-catenin did not affect RhoA-ROCK levels, suggesting that β-catenin was the downstream of RhoA-ROCK regulation. Further data suggested that RhoA increased nuclear translocation of β-catenin and up-regulated β-catenin expression by inhibiting its proteasomal degradation, thereby activating Wnt/β-catenin signaling. Overexpression of β-catenin partially reversed the changes in cell growth, fibrosis and EMT except cell contraction caused by RhoA downregulation. Finally, Y-27632 partially reversed prostatic hyperplasia in vivo, further suggesting the potential of RhoA-ROCK signaling in BPH treatment.

**Conclusion:**

Our novel data demonstrated that RhoA regulated both static and dynamic factors of BPH, RhoA-ROCK-β-catenin signaling axis played an important role in the development of BPH and might provide more possibilities for the formulation of subsequent clinical treatment strategies.

**Supplementary Information:**

The online version contains supplementary material available at 10.1186/s10020-023-00734-2.

## Introduction

Benign prostatic hyperplasia (BPH) is a common histological change characterized by proliferation of prostatic stromal and epithelial cells in the transitional zone (Chughtai et al. 2016), resulting in lower urinary tract symptoms (LUTS) that affect quality of life in men, especially aging men (Kim et al. 2016). The incidence of BPH increases with age, with 50% of the male population having pathologic BPH between 51 and 60 years of age (Platz et al. 2012; Berry et al. 1984), which creates a substantial economic burden (Speakman et al. 2015). Androgen levels, age-related tissue remodeling, inflammatory stimulation, imbalance between cell death and cell proliferation are possible pathogenesis of BPH (Chughtai et al. 2016; Madersbacher et al. 2019). In addition, prostate fibrosis replaces normal tissue with stiff noncompliant fibrotic tissue (Rodriguez-Nieves and Macoska 2013), and EMT leads to accumulation of mesenchymal-like cells derived from the prostatic epithelium (Alonso-Magdalena et al. 2009), both of which are important pathogenesis factors of BPH. Notably, enhanced contraction as well as non-muscle myosin functional activity are also involved in BPH progression (Liu et al. 2023). All of these factors can lead to an increase in prostate volume (static) or tension (dynamic). However, the molecular mechanism of BPH development remains unclear.

Ras homology family member A (RhoA) gene was first isolated from a complementary DNA (cDNA) library from the abdominal ganglia of Aplysia by *Madaule* and *Axel* in the 1980s and named as the rho gene (Madaule and Axel 1985). RhoA plays a regulatory role by cycling between active (GTP-bound) and inactive (GDP-bound) states through guanine nucleotide exchange factors (GEFs) and GTPase activating proteins (GAPs) (Rossman et al. 2005; Tcherkezian and Lamarche-Vane 2007; Buchsbaum 2007). Rho-associated coiled-coil containing kinases (ROCK) (ROCK1 and ROCK2) are the main downstream effector molecules of RhoA and transmit its signal to the downstream pathway through conformation-specific interactions (Leung et al. 1996; Matsui et al. 1996; Jaffe and Hall 2005). RhoA regulates a wide range of cellular functions, such as cell movement, growth, and cytoskeleton. Previous studies have found that RhoA is involved in the development of cardiovascular diseases (Shimokawa et al. 2016), a variety of malignant tumors (Schaefer and Der 2022), and can affect LUTS through Ca^2+^-sensitized mechanisms (Gacci et al. 2011). However, the role of RhoA in BPH remains unclear. Wnt signaling pathways can be divided into β-catenin-dependent (canonical) pathway and non-β-catenin-dependent (non-canonical) pathway. When the canonical pathway is activated, the degradation of β-catenin by the destruction complex is inhibited, the stability of β-catenin is increased and it enters the nucleus to play its role (Doble and Woodgett 2003). In addition to being abnormally activated in prostate cancer (Murillo-Garzon and Kypta 2017), Wnt/β-catenin has also been found to affect EMT of BPH (Chen et al. 2018). It has also been found that the expression of β-catenin in human tissues with BPH is higher than that in normal tissues (Bauman et al. 2014), suggesting that the canonical Wnt/β-catenin pathway may play a role in BPH. RhoA is often considered as the main regulator of non-canonical Wnt/planar cell polarity (PCP) pathway to regulate the cytoskeleton (Fanto et al. 2000; Habas et al. 2001; Tanegashima et al. 2008), but Wnt3a has been found to induce β-catenin accumulation through RhoA (Kim et al. 2017a). It shows that canonical and non-canonical Wnt pathways are not completely independent.

In our current study, we concluded that RhoA was an important factor in the development of BPH. RhoA was found to be up-regulated in hyperplastic human prostate tissue and rat prostate tissue. Further knockdown and overexpression of RhoA in human prostate cell lines identified potential static & dynamic dual roles of RhoA in BPH, including cell proliferation, apoptosis, fibrosis, EMT, and cell contraction. More importantly, we found that RhoA-ROCK and the β-catenin-dependent canonical Wnt pathway constituted a signaling axis that mediated multiple phenotypic changes in the development of BPH. Finally, we found that Y-27632 partially reversed prostatic hyperplasia in rats, suggesting the therapeutic potential of RhoA-ROCK in BPH.

## Materials and methods

### Human prostate tissues collection

Human hyperplastic prostate samples were obtained from 16 male patients requiring radical cystectomy for invasive bladder cancer (mean age 67.3 ± 5.7 years old). Two independent pathologists determined that all samples showed BPH without tumor invasion. Normal prostate samples were obtained from 16 brain-dead men (mean age 29.8 ± 2.4 years old) undergoing organ donation at the Organ Transplant Center of Zhongnan Hospital. Pathological examination showed no hyperplasia. All human samples were obtained with the approval of the Hospital Human Investigation Committee and with the written informed consent of all patients or their relatives. All human researches were conducted in accordance with the principles of the Declaration of Helsinki.

#### Immunofluorescence staining

Human prostate tissues were sectioned into 10 μm thick slices and thawed. The slices were mounted on slides with a cryostat (Leica CM 1850, GER), air-dried and fixed in ice acetone for 10 min, washed in phosphate buffered saline (PBS) and incubated in a PBS mixture supplemented with 0.2% TritonX-100 and 0.1% bovine serum albumin for 2 h. Cells grown on coverslips were fixed in 4% paraformaldehyde for 30 min, washed with PBS, followed by 0.1% TritonX-100 to break the cell membrane, and blocked with goat serum at room temperature for 30 min. After completion of blocking of tissue or cell sections, they were incubated with primary antibodies (listed in Additional file 1: Table S1) overnight, washed with PBS, and incubated with Cy3-labeled or FITC-labeled secondary antibodies (listed in Additional file 2: Table S2) for 1 h. DAPI was used to stain the nucleus. The sections were photographed by fluorescence microscope (Olympus Corporation, JPN).

#### Tissue immunohistochemical (IHC) staining

Human or rat prostate tissue was routinely paraffin embedded after fixation. The samples were cut into 5 μm thick slices, deparaffinized in xylene and dehydrated with graded alcohol. Antigen extraction was carried out in a 10 mM sodium citrate buffer at pH 6.0, heated to 96 °C for 30 min. Endogenous peroxidase activity was quenched by 3% hydrogen peroxide for 15 min. After rinsing and sealing the slides, incubate overnight in the corresponding primary antibody (listed in Additional file 1: Table S1) at 4 ℃. Biotin-conjugated anti-rabbit secondary antibody was incubated for 2 h at 37 °C. The slides were stained with DAB (Solarbio, CHN) and counterstained with Hematoxylin and Eosin Staining Kit (Beyotime, CHN). The results were observed and captured by fluorescence microscope.

### In vitro organ bath studies

Fresh prostate specimens obtained from patients (or brain-dead men) were cut into vertical strips of the same length, approximately 1 × 0.5 × 0.5 cm, and installed lengthwise in a 10 mL organ bath (Multi-Myograph Model 810MS; Danish Myo Technology, DEN). The myograph was connected in line to a PowerLab 4/30 Data Acquisition System (ADInstruments, USA) and in turn to a Dual-Core processor Pentium computer for real-time monitoring of physiological force. The prostate strips were equilibrated at least 1 h in Krebs buffer at 37 °C with continuous bubbling of 95% O_2_ and 5% CO_2_. The buffer was composed of (in mM): NaCl 110, KCl 4.8, CaCl_2_ 2.5, MgSO_4_ 1.2, KH_2_PO_4_ 1.2, NaHCO_3_ 25 and dextrose 11. During the organ bath experiments, the Krebs buffer was changed every 10 min. During the process of equilibration, the tension of the strips was continuously adjusted to 2000 mg. After equilibration, prostate strips were contracted with phenylephrine (PE, Grandpharma, CHN) (10^−5^M). They then were completely washed out and pre-incubated with 10 μM Y-27632 (MedChemExpress, CHN) for 15 min. Then, they were contracted with the same dose of PE. The inhibitory effect of Y-27632 was calculated.

### Cell culture

Human benign prostatic hyperplasia epithelial cell line BPH-1 (Cat. #BNCC339850) was purchased from the Procell Co., Ltd. in Wuhan, China. Human SV40 large-T antigen-immortalized stromal cell line WPMY-1 was purchased from the Stem Cell Bank, Chinese Academy of Sciences in Shanghai, China. Both cell lines had recently been identified. BPH-1 and WPMY-1 cells were cultured in PMI-1640 and Dulbecco's modified Eagle's medium (DMEM, Gibco, USA) containing 10% fetal bovine serum (FBS, Gibco, USA), respectively. Cells were cultured in a humidified atmosphere consisting of 95% air and 5% CO_2_ at 37 °C.

### Cell transfection

RhoA target-specific small interfering RNA (siRNA) was synthesized by Genepharma (CHN), the sequences of each siRNA were shown in the Additional file 3: Table S3. RhoA and β-catenin overexpressed plasmid cDNA were amplified by PCR from cDNA library of human prostate cell line and cloned into 2 × FlagpcDNA3.1 empty vector. Homologous recombinant vector was constructed by one-step method. BPH-1 and WPMY-1 cells were inoculated in 6-well plate and transfected when the cell density reached 50%. The medium in the plate was changed into serum-free medium in advance. 5μL (20 μM) siRNA or 2 μg plasmid was added to 200μL serum-free DMEM, 5μL Lipofectamine 2000 reagent (Invitrogen, USA) was added to 200μL serum-free DMEM. After 5 min, mixed and let stand for 20 min, added the mixture into the corresponding well, and changed to complete medium after 6–8 h. Transfection efficiency was determined by detecting mRNA level of gene by qRT-PCR or protein expression levels by Western blotting.

### Cell counting kit-8 (CCK-8) assay

Cells were seeded in 96-well plate at a density of 2000 per well. The culture medium was changed at 0 h (after cell adhesion), 24 h, 48 h, 72 h, and 96 h, respectively, and CCK-8 reagent (Meilune, CHN) was added to each well 10μL. After incubation in a 37 °C incubator for 1 h, we measured the absorbance at 450 nm with a microplate reader (Thermo Labsystems, USA).

### Flow cytometry analysis

For cell apoptosis analysis, about 1 × 10^6^ cells (including cells in the culture supernatant) were collected. After washing with pre-cooled PBS, the cells were stained with Annexin V-FITC/PI apoptosis Kit (Multi Sciences, CHN), and incubated at 37 ℃ for 5 min away from light, and analyzed by flow cytometry. For cell cycle analysis, the cells were stained with Cell Cycle Staining Kit (Multi Sciences, CHN), and incubated at 37 ℃ for 30 min away from light.

### Cell phalloidine staining

The fixation and membrane breaking of cells were similar to cell immunofluorescence staining. Then the slides were added 50-100μL phalloidine (Servicebio, CHN), incubated at room temperature for 2 h, and washed with PBS. DAPI was used to stain the nucleus. The sections were photographed under a fluorescence microscope.

### Collagen gel contraction assay

The contraction of the cells was evaluated by the collagen gel contraction assay (Cell Biolabs, USA). The treated cells were harvested and resuspended in the culture medium. Collagen, 5X DMEM, neutralizing solution were mixed in the volume ratio of the instructions. Cell suspension and working solution were mixed at 2:8. The 0.5 mL mixture was immediately transferred to each well of the 24-well plate (final cell density: 2 × 10^6^ cells/well) and incubated at 37 °C of 5% CO_2_ for 1 h to polymerize the gel. After gel polymerization, 1 mL of medium was added to the top of the gel and incubation was continued at 37 °C. After 48 h, the gel was separated from the wells using a sterile spatula. Photo measurements were taken at the same time every day and gel contraction was analyzed using Image J software.

### Isolation and extraction of nuclear protein and cytoplasmic protein

According to the kit instructions (Nuclear and Cytoplasmic Protein Extraction Kit, Beyotime, CHN), cells were lysed by protein extraction reagent A with PMSF (Biosharp, CHN) for 15 min, then 10μL reagent B was added to ice bath for 1 min, vortexed and centrifuged, the supernatant was the extracted cytoplasmic protein. For precipitation, the remaining supernatant was completely sucked up, and 50μL nuclear protein extraction reagent added with PMSF was added for precipitation. After 30 min of discontinuous vortex and ice bath, the supernatant obtained by centrifugation was the extracted nuclear protein.

### Protein stability analysis

The protein stability of control and RhoA knockdown cells was analyzed by incubation with 20 μg/ml cycloheximide (CHX) (MedChemExpress, CHN) for the indicated time. The degradation of β-catenin protein was detected by Western blotting analysis.

### Rat prostate tissue samples

A total of 24 specific pathogen-free grade male Sprague–Dawley rats (6 weeks old) weighing 200–250 g were used and randomly divided into 3 groups (n = 8 per group): (1) normal control group (NC), corn oil (MedChemExpress, CHN) injection (subcutaneously (s.c.)) (2) testosterone-induced BPH model group (T-BPH), (T propionate, Sigma-Aldrich, USA) (2 mg/day)/corn oil injection (s.c.) (3) T-BPH + Y group, T (2 mg/day)/corn oil injection (s.c.) +  (Y-27632, MedChemExpress, CHN)/dimethyl sulfoxide (DMSO) (injection into the ventral prostate (v.p.)). Surgeries were performed on day 14, and all procedures were performed under anesthesia by intraperitoneal injection of pentobarbital sodium (35 mg/kg; Abbott Laboratory, USA). A stock solution of Y-27632 was made in DMSO. Rats underwent small midline incisions of the lower abdomen above the penis to expose the ventral prostates. Y-27632 (10 nmoles) in a final volume of 50μL sterile normal saline were injected into both right and left ventral lobes of the prostate with a 30-gauge needle. For the NC and T-BPH groups, 50μL sterile normal saline with nearly commensurable DMSO was injected. After the injection, a 2% lidocaine solution was applied to the wound, and then closed the wound. Rats were euthanized under an intraperitoneal overdose of sodium pentobarbital anesthesia (120 mg/kg) on day 28, and ventral prostates and seminal vesicles were harvested. Animal experiments were conducted at the Animal Center of Zhongnan Hospital of Wuhan University and all animal protocols were approved by the Medical Ethics Committee for Experiments at Zhongnan Hospital of Wuhan University before the experiments were conducted.

### Hematoxylin and Eosin (H&E) staining

Rat prostate tissue samples were fixed with 4% paraformaldehyde with a slice thickness of 5 μm. Tissue sections were stained with hematoxylin–eosin (H&E), the sections were photographed under a fluorescence microscope.

### Total RNA isolation, reverse transcription, and quantitative real-time PCR analysis

The Cell RNA Extraction Kit (Vazyme, CHN) was used to isolate total RNA, and quantification was performed at 260–280 nm using a NanoPhotometer spectrophotometer (Thermo, USA). cDNA was synthesized by reverse transcription of RNA using Hiscript II Q Select RT Kit (Vazyme, CHN). RT-qPCR was performed utilizing the iTaqTM Universal SYBR Green Supermix (Vazyme, CHN). The expression level of gene was normalized to the expression level of GAPDH mRNA, and compared by 2^−ΔΔCT^ method. Primer sequences were listed in the Additional file 4: Table S4.

### Western blotting analysis

Tissues and cells were lysed and ultrasonicated for 30 min in RIPA reagent containing protease inhibitors and phosphatase inhibitors (Sigma-Aldrich, USA). Supernatant was collected after centrifugation at 14,000 g for 10 min. Then, the protein concentration was determined by bicinchoninic acid (BCA) assay. Protein extracts were isolated by sodium dodecyl sulfate/polyacrylamide (SDS/PAGE) gel and transferred to a polyvinylidene fluoride (PVDF, Millipore, USA) membrane using a Bio-RAD wet transfer system. The membrane was blocked at room temperature with Tris-buffered saline containing 0.05% Tween 20 (TBST) buffer of 5% skim milk for 2 h. Primary antibodies (listed in Additional file 1: Table S1) were incubated at 4℃ overnight. After washing for several times, secondary antibodies (listed in Additional file 2: Table S2) were incubated at room temperature for 2 h. These bands were exposed using enhanced chemiluminescence kit (Thermo Scientific Fisher, USA).

### Determination of GTP-bound activated RhoA levels

Tissues were lysed and ultrasonicated for 30 min in RIPA reagent containing protease inhibitors and phosphatase inhibitors. Supernatant was collected after centrifugation at 14,000 g for 10 min. Clarified supernatant was incubated with anti-active RhoA monoclonal antibody (1μL; New East Biosciences, USA) and A/G Agarose bead slurry (20μL) for 1 h at 4 °C. The beads were precipitated by centrifugation, the supernatant was aspirated and discard. Then resuspend the bead pellet in 2X reducing SDS-PAGE sample buffer and boiled for 5 min, centrifuged it at 5000 g for 10 s. The obtained samples were subjected to subsequent Western blotting experiments (The primary and secondary antibodies were listed in Additional file 1: Table S1, Additional file 1: Table S2). Enhanced chemiluminescence kit was used to detect RhoA protein expression.

### Statistical analyses

The results were expressed by the mean ± standard deviation (SD) of at least three independent experiments. Statistical analysis was performed using GraphPadPrism8.0. Student’s t-test was used to determine statistical significance. One-way analysis of variance (ANOVA) was used for multiple group comparisons. Statistical significance was considered as a *p* value < 0.05. Asterisks represent the level of significance: *P < 0.05, **P < 0.01, ***P < 0.001.

## Results

### RhoA-ROCK is up-regulated in human prostate tissues and increases the tension of human prostate strips

**Fig. 1:**
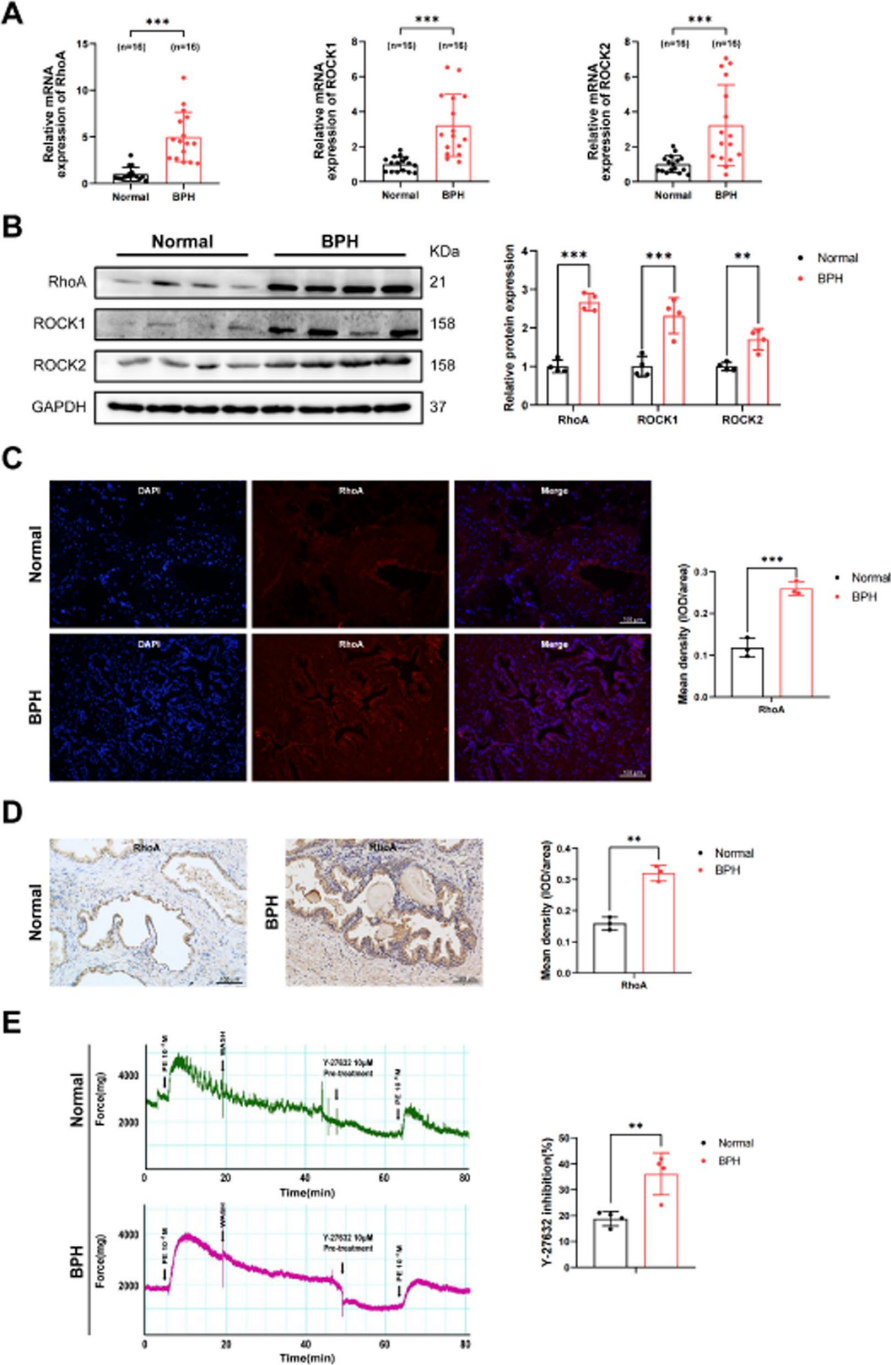
RhoA-ROCK is up-regulated in human prostate tissues and increases the tension of human prostate strips. **A** qRT-PCR analysis demonstrated the mRNA expression level of RhoA, ROCK1 and ROCK2 in normal prostate tissue (n = 16) and BPH tissue (n = 16). **B** Immunoblot assay revealed the protein expression level of RhoA, ROCK1 and ROCK2 in normal prostate tissue and BPH tissue. **C** Immunofluorescence staining of RhoA for normal human prostate and BPH prostate. DAPI (blue) indicated nuclear staining and Cy3-immunofluorescence (red) indicated RhoA protein staining. **D** Immunohistochemical staining of RhoA for normal human prostate and BPH prostate. **E** Representative force tracings of prostate strips from normal human and BPH patients. After 15-min incubation with 10 μM Y-27632, strips were contracted with 10^−5^M PE and the inhibition of Y-27632 was evaluated. The summary graph of inhibition effect of Y-27632 on PE-mediated contraction of human prostate strips (n = 4 different humans for each group). The force for first response to PE was taken as 100% while the inhibition effect of Y-27632 was evaluated as a percentage of this response. Data were expressed as mean ± SD. **p < 0.01, ***p < 0.001

Firstly, we investigated the expression of RhoA-ROCK in clinical samples. The results showed that the mRNA and protein levels of RhoA, ROCK1 and ROCK2 were obviously up-regulated in the hyperplastic prostate tissues compared to the normal prostate tissues (Fig. 1A, B). We found that the expression of active RhoA was up-regulated in hyperplastic prostate tissues, and the ratio of GTP-bound RhoA/Total RhoA was also up-regulated, which further indicated the enhancement of RhoA signal in BPH (Additional file 5: Fig. S1). Immunofluorescence staining was used to evaluate the localization of RhoA in prostate tissue, and it was found that RhoA was distributed in both the human prostate epithelium and stroma (Fig. 1C, D). Therefore, human prostate epithelial cell and stromal cell were used in subsequent experiments. We also examined the expression of Ras homology family member B (RhoB) and Ras homology family member C (RhoC), and found that RhoB was down-regulated while RhoC was up-regulated in BPH (Additional file 5: Fig. S2). We then analyzed the effect of RhoA-ROCK on prostate tissue tension by organ bath studies. ROCK-selective inhibitor Y-27632 pre-treatment could not only lower the baseline tension but also attenuate prostate force generation triggered by PE. Notably, the inhibitory effect of Y-27632 on BPH tissue was more significant when compared with normal tissue (Fig. 1E), indicating the potentiating effect of RhoA-ROCK on prostatic tension.

### RhoA promotes the cell proliferation of WPMY-1 and BPH-1 cells

**Fig. 2:**
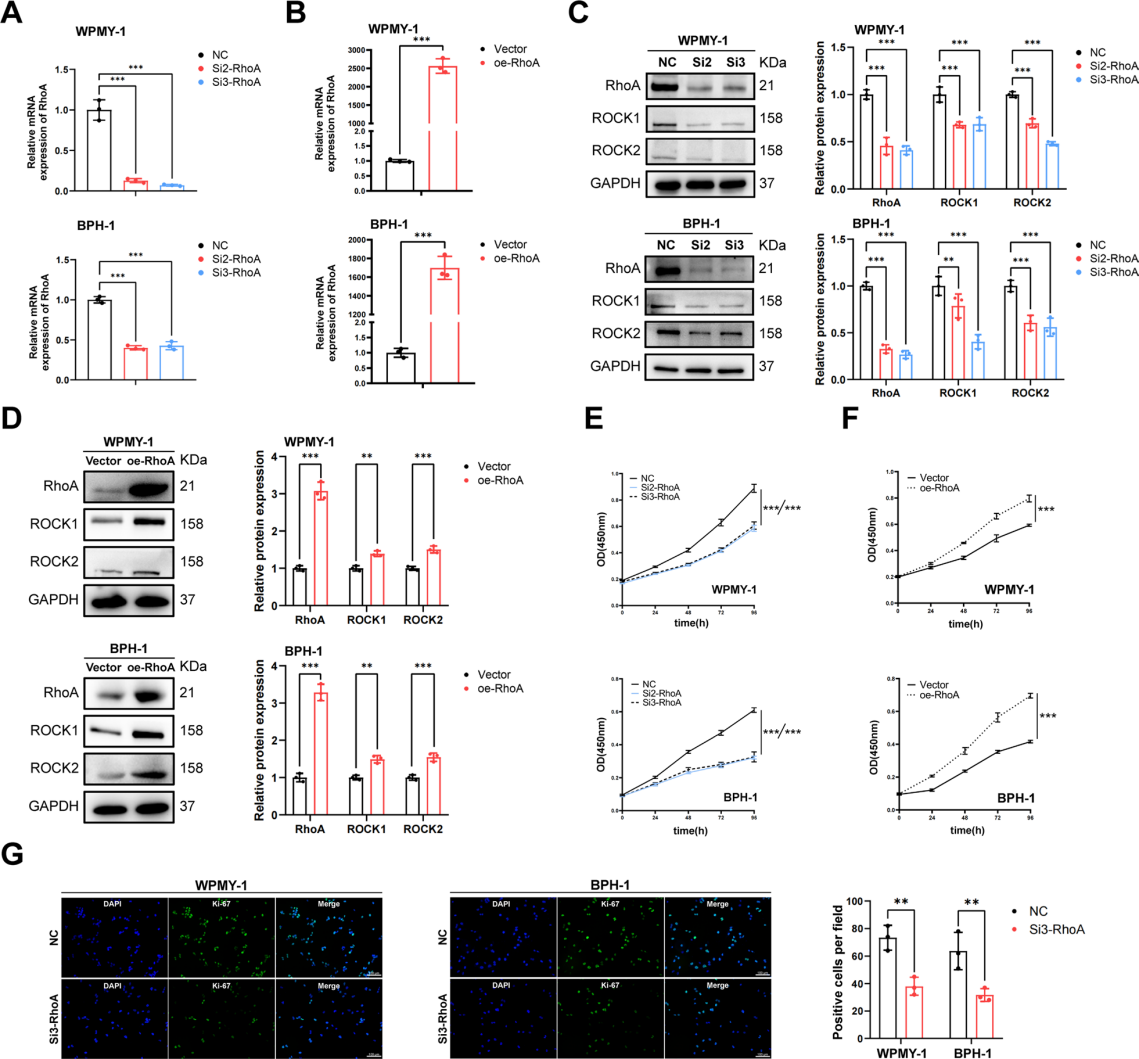
RhoA promotes the proliferation of WPMY-1 and BPH-1 cells. **A** Knockdown efficiency of RhoA at the mRNA levels with two different siRNA sequences in WPMY-1 and BPH-1 cells. **B** Overexpression efficiency of RhoA at the mRNA levels in WPMY-1 and BPH-1 cells. **C** Immunoblot assay of RhoA, ROCK1 and ROCK2 after RhoA knockdown. **D** Immunoblot assay of RhoA, ROCK1 and ROCK2 after RhoA overexpression. **E** The cell proliferation of WPMY-1 and BPH-1 cells measured by CCK-8 assay at different time points after RhoA knockdown. **F** The cell proliferation of WPMY-1 and BPH-1 cells measured by CCK-8 assay at different time points after RhoA overexpression. **G** The Ki-67 staining of WPMY-1 and BPH-1 cells after RhoA knockdown. Data were expressed as mean ± SD. **p < 0.01, ***p < 0.001

RhoA target-specific Si-RNA (Si2-RhoA, Si3-RhoA) and RhoA overexpression plasmids were transfected into WPMY-1 and BPH-1 cells, respectively, to construct cell models of RhoA knockdown and overexpression. The efficiency was confirmed by qRT-PCR (Fig. 2A, B) and western blotting (Fig. 2C, D). The expression of ROCK1 and ROCK2 protein was also down- or up-regulated in accordance with RhoA knockdown or overexpression (Fig. 2C, D). Next, we performed CCK-8 assay to explore the effect of RhoA on proliferation of prostate cell lines. The results showed that down-regulation of RhoA significantly inhibited proliferation of WPMY-1 and BPH-1 cells, while overexpression of RhoA promoted cell proliferation activity (Fig. 2E, F). In addition, Ki-67 immunofluorescence staining showed that RhoA knockdown decreased the Ki-67 positive rate in both cell lines (Fig. 2G).

### RhoA inhibits the cell apoptosis of WPMY-1 and BPH-1 cells, but do not affect the cell cycle

**Fig. 3:**
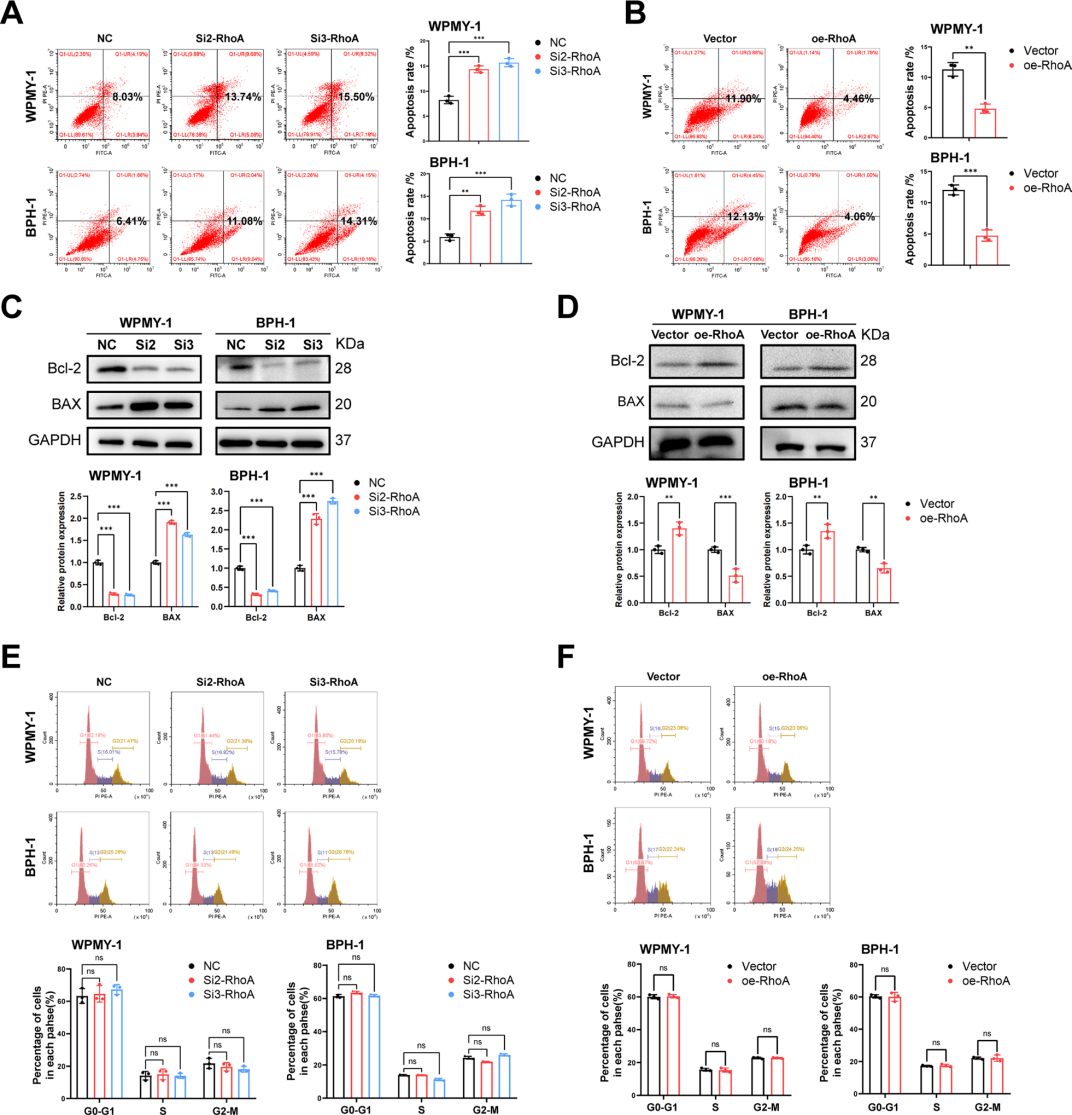
RhoA inhibits the apoptosis of WPMY-1 and BPH-1 cells, but do not affect the cell cycle. **A** Flow cytometry analysis of the cell apoptosis in WPMY-1 and BPH-1 cells after RhoA knockdown. **B** Flow cytometry analysis of the cell apoptosis in WPMY-1 and BPH-1 cells after RhoA overexpression. **C** Immunoblot assay of proteins in relation to cell apoptosis (BAX and Bcl-2) in WPMY-1 and BPH-1 cells after RhoA knockdown. **D** Immunoblot assay of proteins in relation to cell apoptosis in WPMY-1 and BPH-1 cells after RhoA overexpression. **E** Flow cytometry analysis of the cell cycle in WPMY-1 and BPH-1 cells after RhoA knockdown. **F** Flow cytometry analysis of the cell cycle in WPMY-1 and BPH-1 cells after RhoA overexpression. Data were expressed as mean ± SD. ns means no significant difference, **p < 0.01, ***p < 0.001

The effect of RhoA on apoptosis of WPMY-1 and BPH-1 cells was detected by flow cytometry and western blotting. The results showed that down-regulation of RhoA significantly induced cell apoptosis, resulting in decreased Bcl-2 protein expression and increased BAX protein expression (Fig. 3A, C). In contrast, overexpression of RhoA resulted in decreased apoptosis, increased Bcl-2 protein and decreased BAX protein expression (Fig. 3B, D), indicating the inhibitory effect of RhoA on cell apoptosis. However, we found that RhoA did not affect cell cycle progression in both cell lines. The results of flow cytometry showed that either knockdown or overexpression of RhoA did not cause changes in the proportion of cell cycle phases (G0/G1, S, G2/M, Fig. 3E, F).

### RhoA promotes the fibrosis, EMT, cell contration and non-muscle myosin of WPMY-1 and BPH-1 cells

**Fig. 4:**
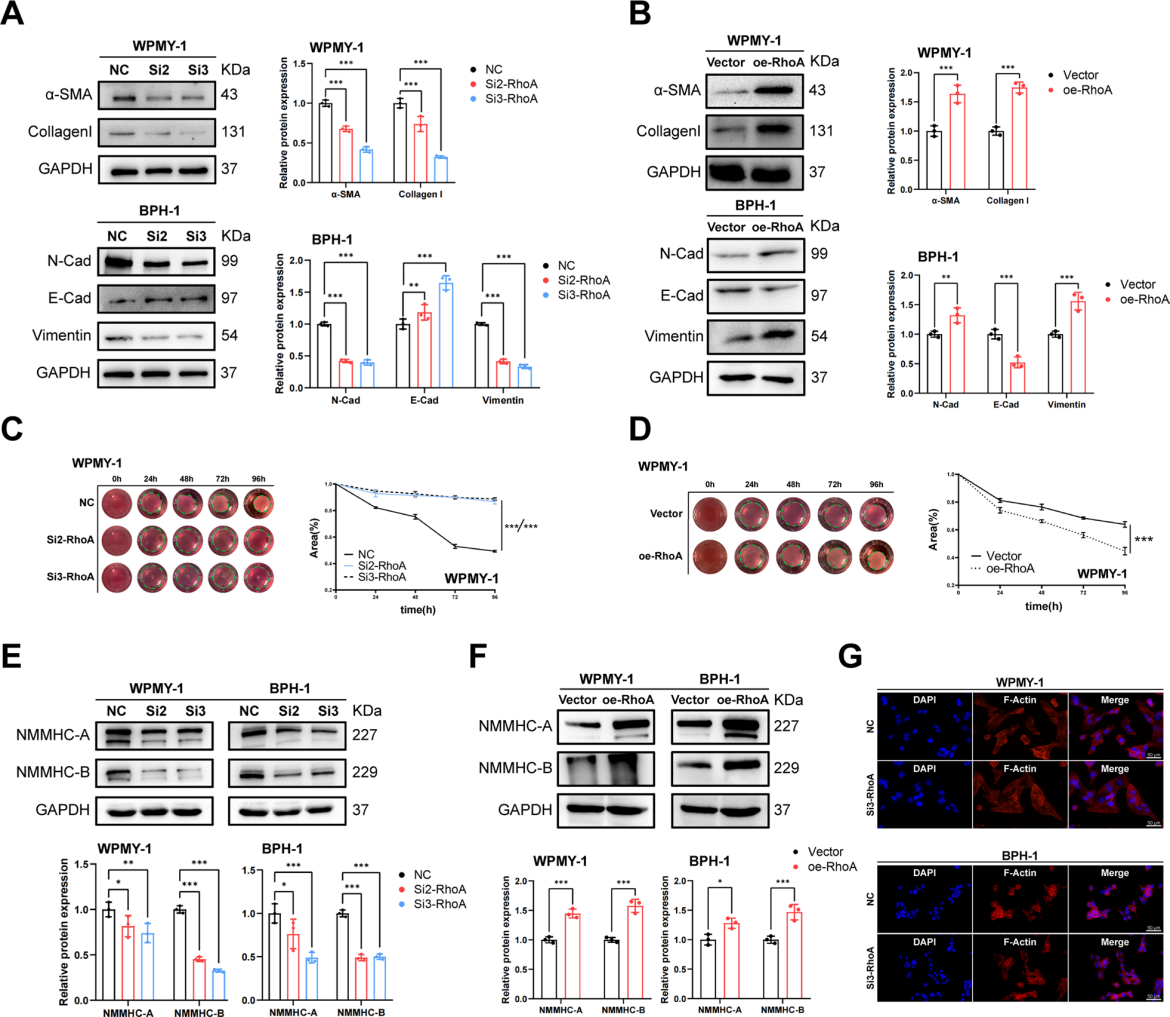
RhoA promotes the fibrosis, EMT, cell contration and non-muscle myosin of WPMY-1 and BPH-1 cells. **A** Immunoblot assay of proteins in relation to cell fibrosis (α-SMA and collagen I) and EMT (E-Cad, N-Cad and Vimentin) in WPMY-1 and BPH-1 cells after RhoA knockdown. **B** Immunoblot assay of proteins in relation to cell fibrosis and EMT in WPMY-1 and BPH-1 cells after RhoA overexpression. **C** The contraction of WPMY-1 cells detected by collagen gel contraction assay at different time points after RhoA knockdown. **D** The contraction of WPMY-1 cells detected by collagen gel contraction assay at different time points after RhoA overexpression. **E** Immunoblot assay of proteins in relation to non-muscle myosin (NMMHC-A and NMMHC-B) in WPMY-1 and BPH-1 cells after RhoA knockdown. **F** Immunoblot assay of proteins in relation to non-muscle myosin in WPMY-1 and BPH-1 cells after RhoA overexpression. **G** The actin cytoskeleton detected by cell phalloidine staining in WPMY-1 and BPH-1 cells after RhoA knockdown. DAPI (blue) indicated nuclear staining, Phalloidine staining (red) indicated F-actin staining. Data were expressed as mean ± SD. *p < 0.05, **p < 0.01, ***p < 0.001

Also, we found that downregulation of RhoA decreased α-SMA and Collagen I expression in WPMY-1 cells, while it decreased N-Cad and Vimentin expression and increased E-Cad expression in BPH-1 cells (Fig. 4A). Overexpression of RhoA had the opposite changes (Fig. 4B), suggesting that RhoA positively regulated prostate fibrosis and EMT. Enhanced contraction as another pathogenic factor of BPH had also been investigated. We found that RhoA knockdown attenuated the contraction of WPMY-1 cells using collagen gel contraction assay (Fig. 4C), while overexpression of RhoA enhanced the contraction of WPMY-1 cells (Fig. 4D). This suggested the potential of RhoA to positively regulate BPH dynamic factor. In addition, RhoA was an important factor in regulating the cytoskeleton, we further investigated the effect of RhoA on the cytoskeleton of BPH-1 and WPMY-1 cells. Western blotting showed that RhoA knockdown reduced the expression of non-muscle myosin IIA and B (Fig. 4E), overexpression of RhoA had the opposite effect (Fig. 4F), and RhoA had no significant effect on the actin cytoskeleton as determined by phalloidin staining (Fig. 4G).

### RhoA-ROCK positively regulates the canonical Wnt/β-catenin signaling pathway

**Fig. 5:**
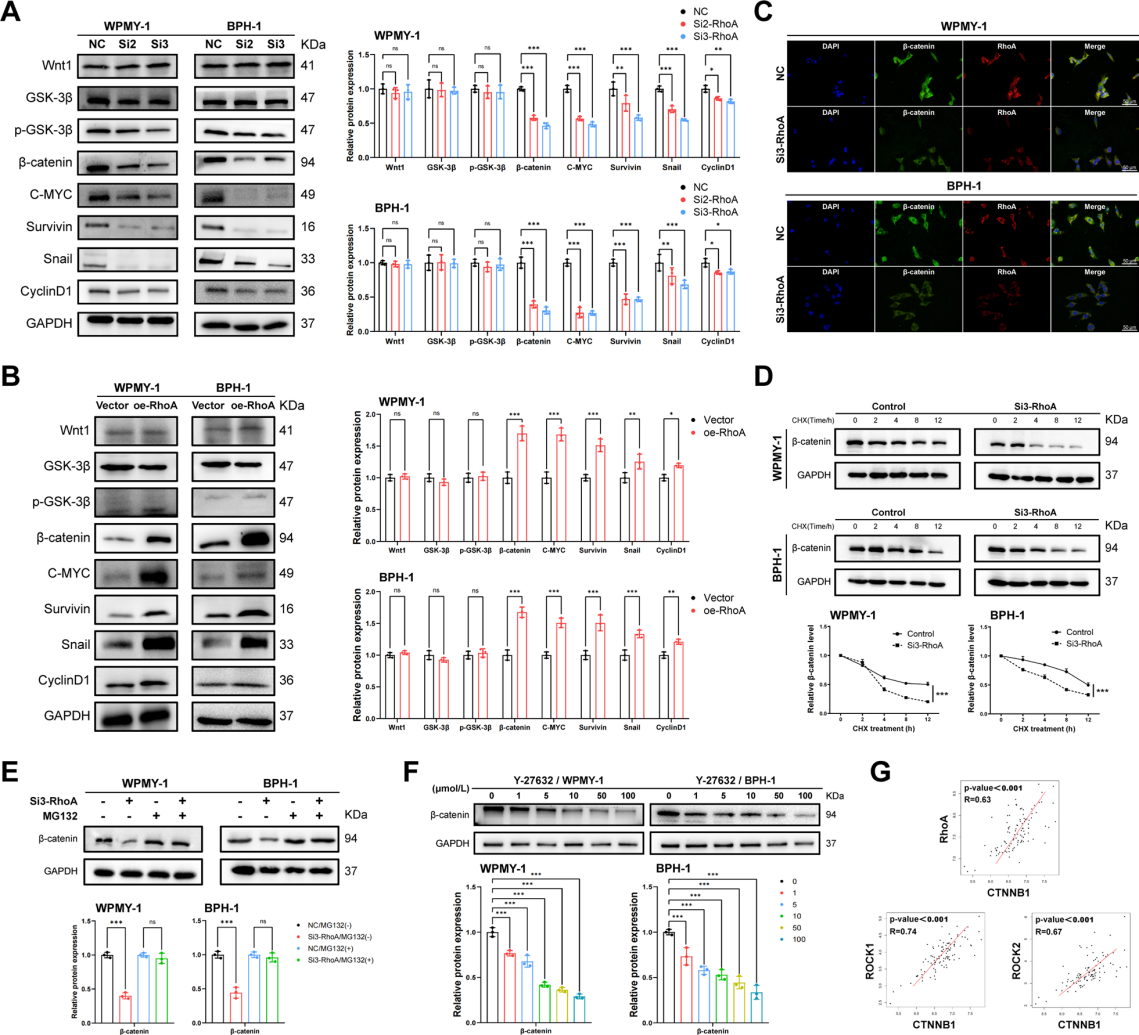
RhoA-ROCK positively regulates the canonical Wnt/β-catenin signaling pathway of WPMY-1 and BPH-1 cells. **A** Immunoblot assay of proteins in relation to canonical Wnt/β-catenin signaling pathway (Wnt1, GSK-3β, p-GSK-3β, β-catenin, C-MYC, Survivin, Snail and CyclinD1) in WPMY-1 and BPH-1 cells after RhoA knockdown. **B** Immunoblot assay of proteins in relation to canonical Wnt/β-catenin signaling pathway in WPMY-1 and BPH-1 cells after RhoA overexpression. **C** Immunofluorescence staining of RhoA and β-catenin for WPMY-1 and BPH-1 cells after RhoA knockdown. DAPI (blue) indicates nuclear staining, FITC-immunofluorescence (green) indicates β-catenin protein staining, and Cy3-immunofluorescence (red) indicates RhoA protein staining. **D** The stability of β-catenin protein determined by immunoblot assay in WPMY-1 and BPH-1 cells after RhoA knockdown. **E** The expression of β-catenin protein after proteasome inhibition determined by immunoblot assay in WPMY-1 and BPH-1 cells. **F** Immunoblot assay of β-catenin in WPMY-1 and BPH-1 cells after different concentrations of Y-27632 treatment for 48 h. **G** GTEx analysis of the correlation between CTNNB1 and RhoA, ROCK1 and ROCK2 in prostate. Data were expressed as mean ± SD. ns means no significant difference, * *p* < 0.05, **p < 0.01, ***p < 0.001

To further explore the molecular mechanism by which RhoA regulated multiple phenotypic changes in prostate cells, we examined the effect of RhoA on Wnt/β-catenin signaling activity. The activation of canonical Wnt/β-catenin signaling was characterized by the increase of β-catenin and its nuclear accumulation. We found that knockdown or overexpression of RhoA had no effect on the protein levels of Wnt1, Glycogen synthase kinase-3β (GSK-3β) and phosphorylated GSK-3β, but knockdown of RhoA significantly down-regulated the expression of β-catenin and its downstream targets C-MYC, Survivin, Snail and CyclinD1. The opposite was observed when RhoA was overexpressed (Fig. 5A, B). Immunofluorescence staining also showed that downregulation of RhoA inhibited β-catenin expression and reduced nuclear accumulation of β-catenin (Fig. 5C). To analyze the reason for the downregulation of β-catenin protein by RhoA knockdown, we treated BPH-1 and WPMY-1 cells with CHX (20 μM, MedChemExpress, CHN), western blotting results showed that the expression of β-catenin in each group gradually decreased with increasing time of CHX treatment. Downregulation of RhoA significantly accelerated the degradation of β-catenin. The proteasome inhibitor MG132 (5 μM, MedChemExpress, CHN) blocked the decrease in β-catenin expression caused by RhoA knockdown (Fig. 5E), indicating that the degradation of β-catenin through proteasome pathway could be inhibited by RhoA. Furthermore, we treated WPMY-1 and BPH-1 cells with different concentrations of Y-27632 and found that inhibition of ROCK also down-regulated β-catenin expression and the effect was concentration-dependent (Fig. 5F). Next, we downloaded RNA-seq data (TPM) from The Genotype-Tissue Expression Project (GTEx) database, performed log2 transformation on gene expression, and performed Spearman correlation analysis by R language. We found that in human prostate, CTNNB1 (β-catenin encoding gene) was positively correlated with the expression of RhoA, ROCK1 and ROCK2 (Fig. 5G).

### RhoA-ROCK promotes cell proliferation, fibrosis and EMT, inhibits cell apoptosis of WPMY-1 and BPH-1 cells by regulating β-catenin

**Fig. 6:**
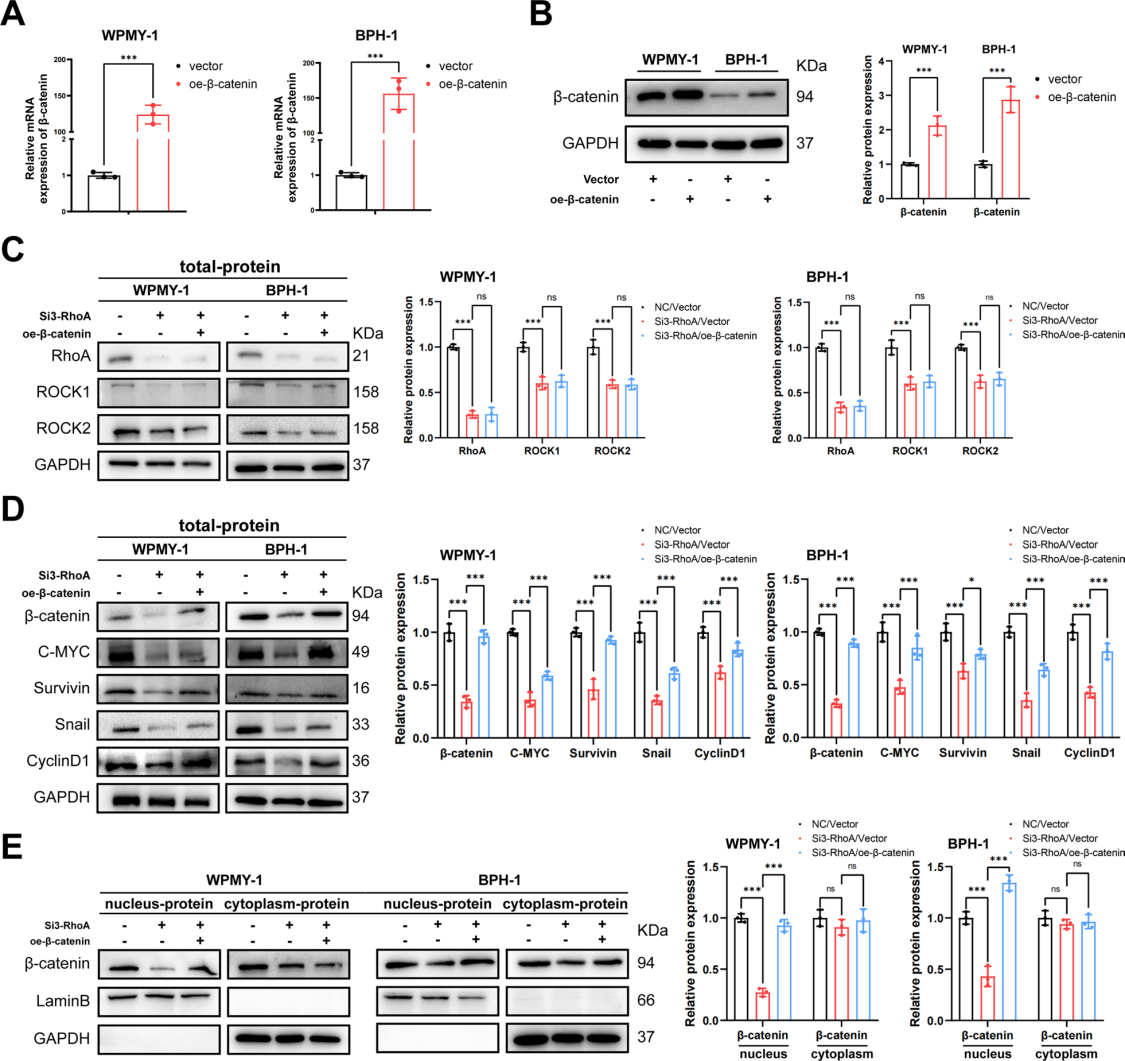
Verification of co-transfection system and β-catenin nuclear translocation analysis in WPMY-1 and BPH-1 cells. **A** Overexpression efficiency of β-catenin at mRNA levels in BPH-1 and WPMY-1 cells. **B** Overexpression efficiency of β-catenin at protein levels in BPH-1 and WPMY-1 cells. **C** Immunoblot assay of RhoA, ROCK1 and ROCK2 in WPMY-1 and BPH-1 cells after co-transfection. **D** Immunoblot assay of Wnt/β-catenin downstream proteins (β-catenin, C-MYC, Survivin, Snail and CyclinD1) in WPMY-1 and BPH-1 cells after co-transfection. **E** Immunoblot assay of nucleus-proteins and cytoplasm-proteins (β-catenin) in WPMY-1 and BPH-1 cells after co-transfection. Data were expressed as mean ± SD. ns means no significant difference, *p < 0.05, ***p < 0.001

**Fig. 7:**
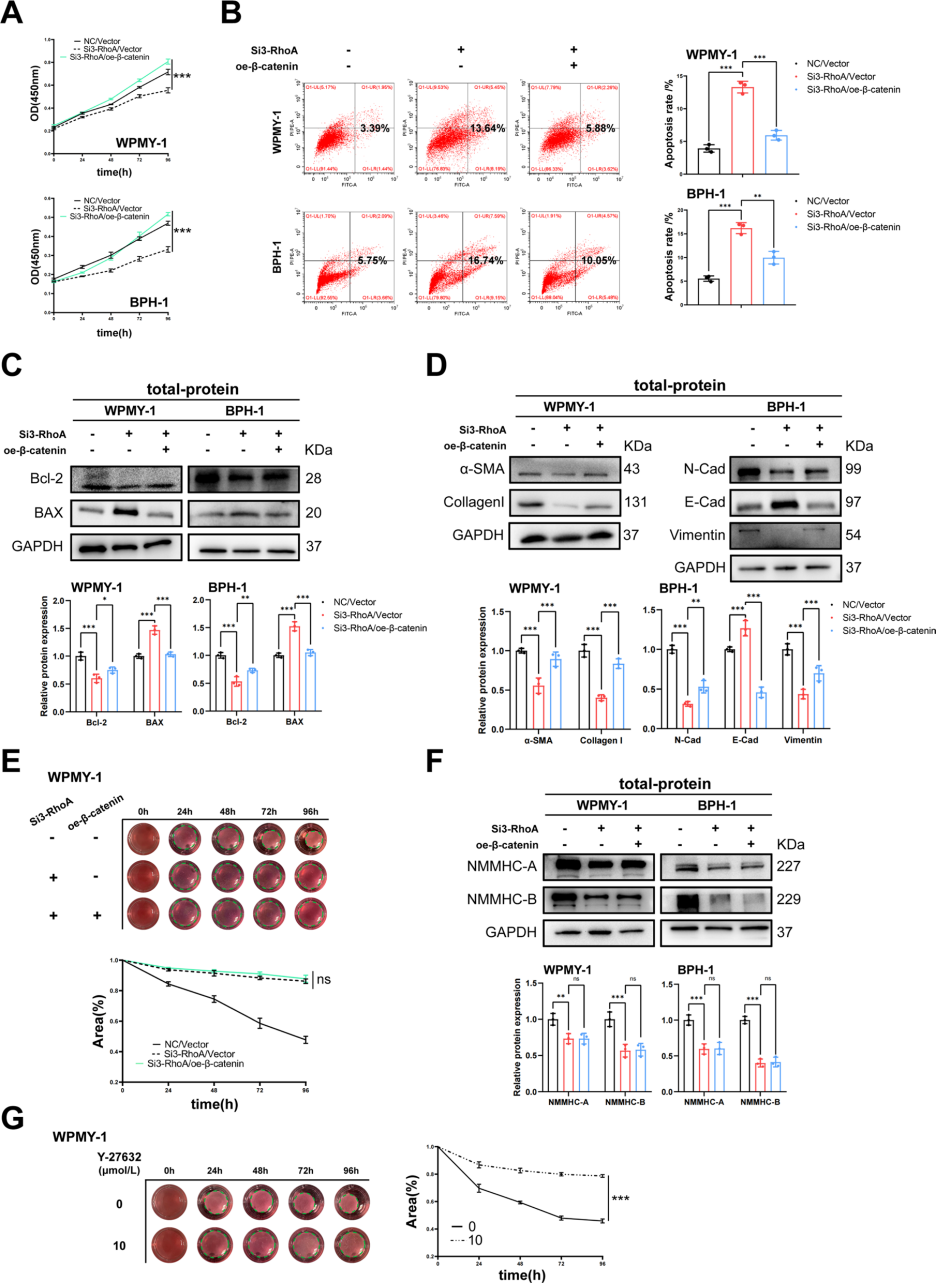
RhoA-ROCK promotes cell proliferation, fibrosis and EMT, inhibits cell apoptosis of WPMY-1 and BPH-1 cells by regulating β-catenin. **A** The cell proliferation of WPMY-1 and BPH-1 cells measured by CCK-8 assay at different time points after co-transfection. **B** Flow cytometry analysis of the cell apoptosis in WPMY-1 and BPH-1 cells after co-transfection. **C** Immunoblot assay of proteins in relation to cell apoptosis (BAX and Bcl-2) in WPMY-1 and BPH-1 cells after co-transfection. **D** Immunoblot assay of proteins in relation to fibrosis (α-SMA and collagen I) and EMT (E-Cad, N-Cad and Vimentin) in WPMY-1 and BPH-1 cells after co-transfection. **E** The contraction of WPMY-1 cells detected by collagen gel contraction assay at different time points after co-transfection. **F** Immunoblot assay of proteins in relation to non-muscle myosin (NMMHC-A and NMMHC-B) in WPMY-1 and BPH-1 cells after co-transfection. **G** The contraction of WPMY-1 cells detected by collagen gel contraction assay at different time points after 10 μM Y-27632 treatment. Data were expressed as mean ± SD. ns means no significant difference, * *p* < 0.05, **p < 0.01, ***p < 0.001

A co-transfection system of β-catenin overexpressed plasmid and RhoA SiRNA3 was constructed, the transfection efficiency of β-catenin plasmid in BPH-1 and WPMY-1 cells was determined by qRT-PCR and western blotting (Fig. 6A, B). Co-transfection of cells revealed that transfection of Si-RhoA significantly down-regulated the protein expression of RhoA, ROCK1, and ROCK2, consistent with the previous results, while simultaneous overexpression of β-catenin did not cause changes in them (Fig. 6C). Overexpression of β-catenin significantly reversed the decrease of Wnt/β-catenin downstream proteins caused by RhoA down-regulation (Fig. 6D), which confirmed the successful construction of the co-transfected cell model. We further isolated the nuclear and cytoplasm proteins of BPH-1 and WPMY-1 cells and found that the expression of β-catenin in the nucleus decreased by RhoA downregulation, and the level of nuclear β-catenin protein in the co-transfection group was recovered. However, cytoplasmic β-catenin protein was not significantly changed (Fig. 6E). Next, we performed a series of rescue experiments to investigate whether RhoA-mediated phenotype changes in prostate cell lines were through β-catenin. CCK-8, flow cytometry and western blotting revealed that the overexpression of β-catenin reversed RhoA knockdown triggered cell proliferation decrease and cell apoptosis increase, accompanied by the corresponding changes in Bcl-2 and BAX proteins (Fig. 7A–C). Additionally, the aforementioned fibrosis-related proteins (α-SMA and Collagen I) and EMT-related proteins (N-Cad, E-Cad and Vimentin) changed by the RhoA knockdown were all reversed by β-catenin overexpression (Fig. 7D). However, β-catenin neither reverse the contraction of WPMY-1 cells inhibited by RhoA downregulation, nor affected the downregulation of non-muscle myosin IIA and B induced by the Si-RhoA (Fig. 7E, F). We also focused that in addition to Si-RhoA, Y-26732 also significantly inhibited cell contraction (Fig. 7G), suggesting that RhoA-ROCK caused contraction in WPMY-1 cells, but probably independently of β-catenin levels.

### Inhibition of ROCK partially reverses prostatic hyperplasia of the BPH rat model.

**Fig. 8:**
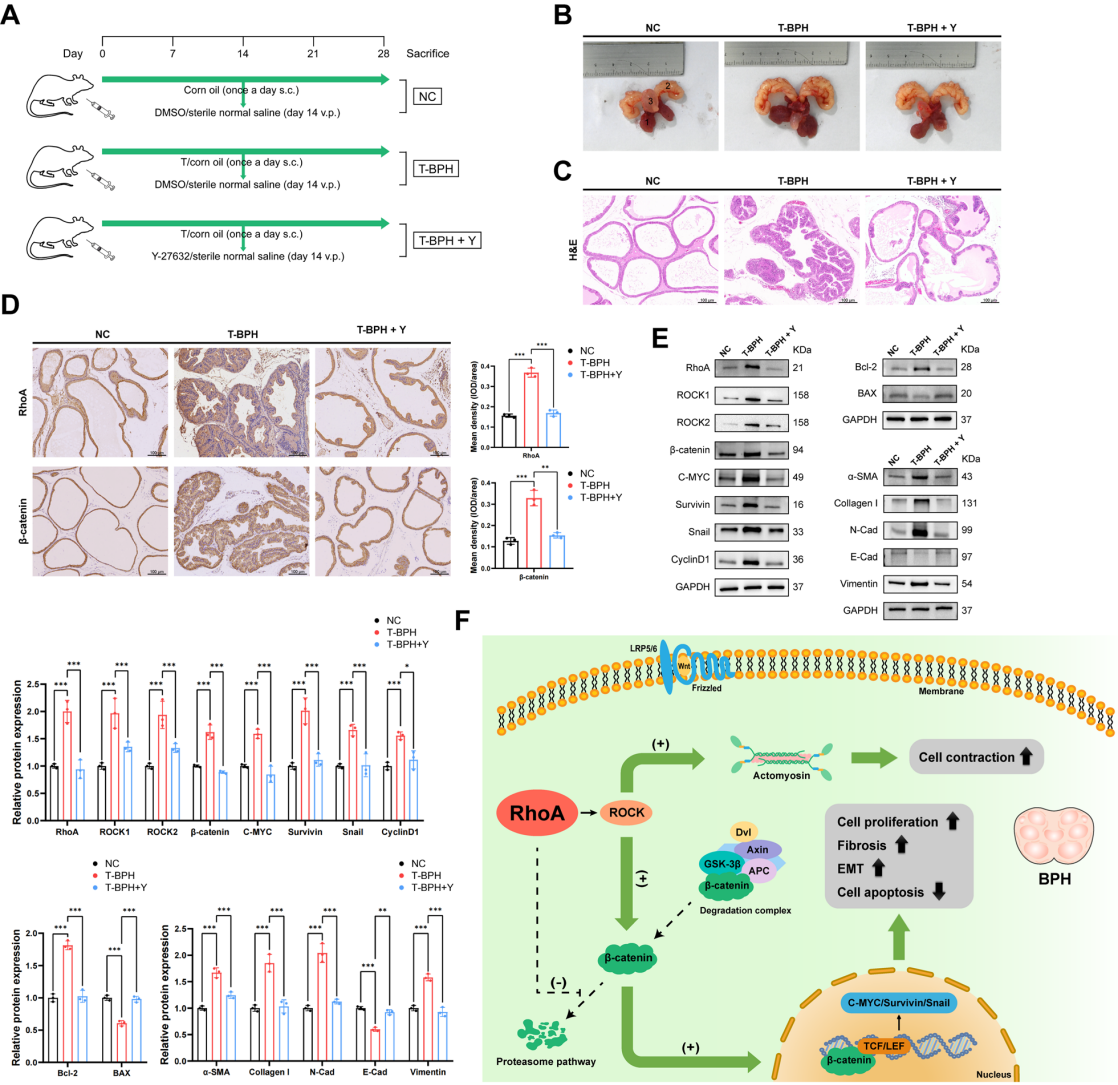
Inhibition of ROCK partially reverses prostatic hyperplasia of the BPH rat model. **A** The simple schematic model of in vivo experiments. **B** The rat urogenital tissues from NC, T-BPH, and T-BPH + Y rats. (1) ventral prostate, (2) seminal vesicle, and (3) bladder. (**C**) Representative H&E staining of NC, T-BPH, and T-BPH + Y rat prostates. **D** Representative Immunohistochemical staining of RhoA and β-catenin for NC, T-BPH, and T-BPH + Y rat prostates. **E** Immunoblot assay of proteins in relation to RhoA-ROCK-β-catenin signal axis system (RhoA, ROCK1, ROCK2, β-catenin, C-MYC, Survivin, Snail and CyclinD1) and related phenotypes in NC, T-BPH, and T-BPH + Y rat prostates. **F** The simple schematic model proposed for the central roles of RhoA-ROCK-β-catenin signal axis system in the development of BPH. Data were expressed as mean ± SD. *p < 0.05, **p < 0.01, ***p < 0.001

The testosterone-induced BPH (T-BPH) rat model was validated by the increased volume of the ventral prostate and by H&E staining (Fig. 8B, C). IHC staining indicated that RhoA and β-catenin expression was higher in T-BPH rats, especially in the epithelial components (Fig. 8D). Western blotting showed that the protein levels of the RhoA-ROCK-β-catenin signal axis and Phenotypic associated protein (Bcl-2, α-SMA, Collagen I, N-Cad and Vimentin) increased to various degrees for T-BPH rat prostates, while BAX and E-Cad decreased. (Fig. 8E). The prostates of T-BPH rats treated with Y-27632 had a certain degree of therapeutic effect on the organ morphology and histological morphology, and the expression of related proteins had been reversed to varying degrees. A schematic representation for the role of RhoA-ROCK-β-catenin signaling axis in the development of BPH is shown at the end (Fig. 8F).

## Discussion

BPH is a very common male disease and a major urological challenge. There is an urgent need to identify new molecular regulatory mechanisms and therapeutic targets. In this study, we found that RhoA played dynamic & static dual roles in the development of BPH. Our results suggested that RhoA-ROCK regulated prostate cell contraction on the one hand, and regulated the downstream genes, including C-MYC, Survivin and Snail, via the RhoA-ROCK-β-catenin signaling axis to affect cell proliferation, apoptosis, fibrosis and EMT on the other hand.

Rho GTPases constitute a distinct family in the Ras-related small GTPases superfamily, and RhoA, as a representative of them, plays an important role in different diseases. Our study found that RhoA-ROCK was up-regulated in BPH human tissues, and ROCK inhibition could significantly inhibit the contraction of prostate tissue strips. RhoA-GTP, which activated downstream effector molecules such as ROCK, was also found to be up-regulated in BPH. Interestingly, here we found that RhoC was up-regulated in BPH, whereas RhoB was down-regulated. A previous study of endothelial cell morphology found that RhoB negatively regulated RhoA (Howe and Addison 2012), which was consistent with the trend we observed in human prostate. RhoA regulated a wide variety of cell functions and its effects on cell growth had been extensively studied. RhoA enhanced cell proliferation in human endometrial epithelial cells (EECs) (Huang et al. 2020). RhoA also mediated the regulatory effect of vitamin D receptor agonist Elocalcitol and Upregulated gene 11 (URG11) on cell proliferation in prostate cells (Penna et al. 2009; Zhang et al. 2018). It had been speculated that active RhoA promoted the formation of actin contraction rings on the equatorial membrane to affect the cell division process (Basant and Glotzer 2018). Consistent with these studies, we demonstrated the positive regulatory effect of RhoA on the proliferation of human prostate cell lines. In addition, our study demonstrated that RhoA knockdown could promote apoptosis of WPMY-1 and BPH-1 cells. Parallelly, the apoptosis associated gene BAX was increased while Bcl-2 was decreased. This was similar to the results of previous studies on apoptosis induced by RhoA knockdown in lung cancer cells and zebrafish embryogenesis (Liu et al. 2017; Zhu et al. 2008). In contrast, RhoA had previously been reported to trigger apoptosis by regulating actin cytoskeleton as a concentrated signal (Gajate and Mollinedo 2005; Papadopoulou et al. 2008; Ohgushi et al. 2010; Zhang et al. 2011). Our study showed that RhoA knockdown did not significantly change the actin cytoskeleton of prostate cell lines. We speculated that this might be due to the fact that the balance of F-actin regulated by the combined mammalian diaphanous-related formin (mDia1) and ROCK signaling pathways remained stable and the regulation of actin cytoskeleton depended on cell type, cell background and microenvironment.

Human prostatic hyperplasia is mainly stromal hyperplasia. Stromal fibrosis leads to decreased urinary compliance and increased prostate passive tension. EMT exacerbates the accumulation of mesenchymal-like cells. A previous study found that reduced expression of ROCK1 or ROCK2 was sufficient to protect mice from experimental pulmonary fibrosis (Knipe et al. 2018). RhoA had also been found to be involved in the pathological processes of liver fibrosis and silicosis fibrosis (Cai et al. 2020; Li et al. 2019). Our results showed that RhoA knockdown down-regulated the expression of α-SMA and Collagen I in WPMY-1 cells, and RhoA overexpression reversed the effect. In addition, silent RhoA could attenuate the expression of N-Cad and Vimentin, and enhance the expression of E-Cad in BPH-1 cells, while overexpression of RhoA reversed these EMT markers, suggesting the potential anti-fibrosis and EMT effects of RhoA downregulation. A series of in vitro experiments of Y-27632 had been showed the effect of RhoA-ROCK on prostate contraction (Rees et al. 2003; Lam et al. 2013). Here, we studied the direct effect of RhoA on prostate cell contraction, and found that RhoA positively regulated WMPY-1 cell contraction. In addition, RhoA knockdown resulted in down-regulated expression of cytoskeletal components NMMHC-A and NMMHC-B. Previous studies had shown that NMMHC-A and NMMHC-B could promote proliferation and inhibit apoptosis of prostate cell lines (He et al. 2021), their downregulation was therefore consistent with the observed proliferation and apoptosis trends.

After clarifying the role of RhoA in the development of BPH, we further explored the potential regulatory mechanism. In the absence of Wnt signaling, the degradation complex containing adenomatous polyposis coli (APC), GSK-3β and AXIN is phosphorylated and targets β-catenin for ubiquitination and proteasome degradation. At the same time, members of the Transcription factor (TCF)/ Lymphoid enhancer factor (LEF) family associate in a repressive complex with transducing-like enhancer protein (TLE) co-repressor proteins to inhibit β-catenin target genes. In current study, we revealed that RhoA knockdown led to downregulation of β-catenin and several important target genes of canonical Wnt/β-catenin pathway, including C-MYC, Survivin, Snail and CyclinD1. RhoA overexpression up-regulated the expression of these proteins. C-MYC was a proto-oncogene that was highly expressed in high-grade prostate tumors and could accelerate the growth of prostate cancer (Nagy et al. 2009; Wu et al. 2021; Buttyan et al. 1987). It was worth noting that C-MYC was also known to be an important factor regulating RhoA transcriptional regulation. It was found that RhoA could enhance the expression of C-MYC by activating NF-κB or β-catenin (Kim et al. 2017a, 2017b), enhance the stability and transcriptional activity of C-MYC by ROCK1 (Zhang et al. 2014), or enhance the activity of C-MYC through phosphorylation of p300 (Acetyltransferase p300) by ROCK2 (Tanaka et al. 2006). C-MYC could promote the transcription of RhoA, thus forming a positive feedback loop, which was consistent with our results. Survivin was associated with apoptosis and was over expressed in hyperplastic prostate (Shariat et al. 2005). Snail was involved in EMT in a variety of diseases and had been found to mediate EMT in BPH (Zhang et al. 2021; Tang et al. 2020; Kim et al. 2021). The changes of these downstream genes were consistent with the phenotypic function regulated by RhoA. The changes of CyclinD1 did not lead to significant changes in cell cycle, which might be related to the fact that the positive and negative balance of the Cyclin-cyclin dependent kinase (CDK)-cyclin dependent kinase inhibitor (CKI) signal regulatory network was not broken. We further found that β-catenin was co-localized with RhoA in WPMY-1 and BPH-1 cells, and RhoA enhanced the stability of β-catenin protein, reduced the proteasome pathway degradation of β-catenin, as well as promoted its nuclear translocation. In a previous study of aging-related bone loss, active RhoA initiated a series of signals that ultimately destabilized β-catenin (Shi et al. 2021). We speculated that different tissues or cell types might be the reason for the different relationships between RhoA and β-catenin. We further constructed a co-transfection system of β-catenin overexpression plasmid and RhoA small interfering RNA in prostate cell lines. Using this system, we demonstrated that β-catenin overexpression partially reversed RhoA knockdown induced cell proliferation, fibrosis, and EMT inhibition, as well as increased apoptosis. Markers of the aforementioned biological processes were correspondently recovered. Notably, overexpression of β-catenin reversed the expression of downstream target genes, but did not change the protein expression of RhoA-ROCK, which confirmed that β-catenin was located in the downstream of RhoA-ROCK. In addition, overexpression of β-catenin did not significantly alter cell contraction or non-muscle myosin II expression, suggesting that RhoA-ROCK might regulate contraction via other pathways. Finally, we translated our in vitro study to an in vivo study. The expression of RhoA-ROCK-β-catenin axis-related proteins was up-regulated in constructed T-BPH rat model. Y-27632 showed therapeutic effect on T-BPH rats and reversed the expression of pathway and phenotypic proteins. There were still some limitations in this study. Testosterone induced prostatic epithelial hyperplasia, considering that human prostatic hyperplasia mainly occurred in stroma, animal models more suitable for human physiological conditions should be investigated. The use of ROCK inhibitor could not fully mimic the effects of RhoA downregulation in vivo, transgenic mice will further confirm the role of RhoA in the pathogenesis of BPH. These will be further supplemented in future studies.

## Conclusions

In summary, we found that RhoA regulated the static and dynamic dual factors of BPH, including cell proliferation, apoptosis, fibrosis, EMT and contraction. There was a crosstalk between RhoA-ROCK and canonical Wnt/β-catenin signaling pathway in human prostate cell lines. The RhoA-ROCK-β-catenin signaling axis affected multiple progresses in the development of BPH, providing potential therapeutic targets for patients with BPH.

### Supplementary Information

**Additional file 1: Table S1**. List of primary antibodies.

**Additional file 2: Table S2. **List of secondary antibodies.

**Additional file 3: Table S3. **Sequences of each siRNA.

**Additional file 4: Table S4. **Primer sequences used for qRT-PCR.

**Additional file 5:** **Figure S1. **Immunoblot assay revealed the protein expression level of GTP-RhoA and Total RhoA in normal prostate tissue and BPH tissue. Data were expressed as mean±SD. * *p*＜0.05. **Figure S2. **Expression of RhoB and RhoC in human prostate tissue.

## Data Availability

The data used to support the findings of the present study are available from the corresponding author upon reasonable request.

## Abbreviations

APC: Adenomatous polyposis coli
BCA: Bicinchoninic acid
BPH: Benign prostatic hyperplasia
CCK-8: Cell counting kit-8
CDK: Cyclin dependent kinase
cDNA: Complementary DNA
CHX: Cycloheximide
CKI: Cyclin dependent kinase inhibitor
EECs: Endometrial epithelial cells
EMT: Epithelial–mesenchymal transition
FBS: Fetal bovine serum
GAPs: GTPase activating proteins
GEFs: Guanine nucleotide exchange factors
GSK-3β: Glycogen synthase kinase-3β
IHC: Immunohistochemical
LEF: Lymphoid enhancer factor
LUTS: Lower urinary tract symptoms
mDia1: Mammalian diaphanous-related formin
mRNA: Messenger RNA
p300: Acetyltransferase p300
PBS: Phosphate-buffered saline
PCP: Planar cell polarity
PCR: Polymerase chain reaction
PE: Phenylephrine
PVDF: Polyvinylidene fluoride membrane
qRT-PCR: Quantitative real-time PCR
RhoA: Ras homology family member A
RhoB: Ras homology family member B
RhoC: Ras homology family member C
ROCK: Rho-associated coiled-coil containing kinase
SDS/PAGE: Sodium dodecyl sulfate/polyacrylamide gel
siRNA: Small interfering RNA
T-BPH: Testosterone-induced BPH model group
TCF: Transcription factor
TLE: Transducing-like enhancer protein
URG11: Upregulated gene 11
