# Supplementary material for: Mechanism of RhoA regulating benign prostatic hyperplasia: RhoA-ROCK-β-catenin signaling axis and static & dynamic dual roles
Source: Mol Med. 2023 Oct 20;29:139. doi: 10.1186/s10020-023-00734-2 (PMC10589999; doi:10.1186/s10020-023-00734-2)
Supplement: Supplementary file 1 — Additional file 1: Table S1. List of primary antibodies. [file 10020_2023_734_MOESM1_ESM.docx]

**Additional file 1: Table S1: List of primary antibodies**

| Antigens | Species antibodies raised in | Dilution used | Supplier |
| --- | --- | --- | --- |
| RhoA | Rabbit monoclonal | 1:5000(WB)  1:150(IF)  1:150(IHC) | abcam  Ab187027 |
| RhoB | Rabbit polyclonal | 1:1000(WB)  1:100(IF)  1:100(IHC) | Affinity  DF4438 |
| RhoC | Rabbit polyclonal | 1:1000(WB)  1:100(IF)  1:100(IHC) | Affinity  DF6207 |
| ROCK1 | Rabbit monoclonal | 1:500(WB) | abcam  Ab134181 |
| ROCK2 | Rabbit monoclonal | 1:10000(WB) | abcam  Ab125025 |
| Ki-67 | Rabbit polyclonal | 1:1200 (IF) | Servicebio  GB111141 |
| Bcl-2 | Mouse monoclonal | 1:1000(WB) | Proteintech  68103-1-Ig |
| BAX | Rabbit polyclonal | 1:1000(WB) | Abclonal  A12009 |
| α-SMA | Mouse monoclonal | 1:1000(WB) | Servicebio  GB13044 |
| Collagen I | Rabbit polyclonal | 1:1000(WB) | Abclonal  A1352 |
| N-Cad | Mouse monoclonal | 1:1000(WB) | Proteintech  66219-1-Ig |
| E-Cad | Rabbit polyclonal | 1:1000(WB) | Proteintech  20874-1-AP |
| Vimentin | Rabbit monoclonal | 1:1000(WB) | Abclonal  A19607 |
| NMMHC-A(MYH9) | Rabbit polyclonal | 1:1000(WB) | Abclonal  A16923 |
| NMMHC-B(MYH10) | Rabbit polyclonal | 1:1000(WB) | Abclonal  A12029 |
| Wnt | Rabbit polyclonal | 1:1000(WB) | Proteintech  27935-1-AP |
| β-catenin | Mouse monoclonal | 1:5000(WB)  1:200(IF)  1:2500(IHC) | Proteintech  66379-1-Ig |
| GSK-3β | Rabbit monoclonal | 1:1000(WB) | HUABIO  ET1607-71 |
| p-GSK-3β | Rabbit monoclonal | 1:1000(WB) | HUABIO  ET1607-60 |
| C-MYC | Mouse monoclonal | 1:1000(WB) | Proteintech  67447-1-Ig |
| Survivin | Rabbit polyclonal | 1:1000(WB) | Affinity Biosciences  AF0617 |
| Snail | Rabbit polyclonal | 1:1000(WB) | Abclonal  A11794 |
| CyclinD1 | Mouse monoclonal | 1:1000(WB) | Proteintech  60186-1-Ig |
| LaminB | Rabbit polyclonal | 1:1000(WB) | Abclonal  A1910 |
| GAPDH | Rabbit polyclonal | 1:10000(WB) | Abclonal  AC027 |
