# Supplementary material for: Mechanism of RhoA regulating benign prostatic hyperplasia: RhoA-ROCK-β-catenin signaling axis and static & dynamic dual roles
Source: Mol Med. 2023 Oct 20;29:139. doi: 10.1186/s10020-023-00734-2 (PMC10589999; doi:10.1186/s10020-023-00734-2)
Supplement: Supplementary file 2 — Additional file 2: Table S2. List of secondary antibodies. [file 10020_2023_734_MOESM2_ESM.docx]

**Additional file 2: Table S2: List of secondary antibodies**

| Secondary detection system used | Host | Dilution used | Supplier |
| --- | --- | --- | --- |
| HRP-conjugated Affinipure Goat Anti-Mouse IgG(H+L) | Goat | 1:10000 (WB) | Proteintech  SA00001-1 |
| HRP-conjugated Affinipure Goat Anti-Rabbit IgG(H+L) | Goat | 1:10000 (WB) | Proteintech  SA00001-2 |
| Anti-rabbit IgG (H+L), F(ab')2 fragment (Alexa Fluor® 488 Conjugate) | Goat | 1:50 (IF) | Cell Signaling Technology, USA, cat. no. 4412 |
| Anti-mouse IgG (H+L), F(ab')2 Fragment (Alexa Fluor® 488 Conjugate) | Goat | 1:50 (IF) | Cell Signaling Technology, USA, cat. no. 4408 |
| Hoechst 33342 (1 mg/ml) nucleic acid staining (DAPI) | - | 1:750 (IF) | Molecular Probes/Invitrogen, Carlsbad, CA, USA, cat. no. A11007 |
