# Supplementary material for: Mechanism of RhoA regulating benign prostatic hyperplasia: RhoA-ROCK-β-catenin signaling axis and static & dynamic dual roles
Source: Mol Med. 2023 Oct 20;29:139. doi: 10.1186/s10020-023-00734-2 (PMC10589999; doi:10.1186/s10020-023-00734-2)
Supplement: Supplementary file 3 — Additional file 3: Table S3. Sequences of each siRNA. [file 10020_2023_734_MOESM3_ESM.docx]

**Additional file 3: Table S3: Sequences of each siRNA**

| siRNA | Number | Sequence (5’ to 3’) |
| --- | --- | --- |
| siRNA 1 | RHOA-homo-628 | GAAGGAUCUUCGGAAUGAUTT |
|  |  | AUCAUUCCGAAGAUCCUUCTT |
| siRNA 2 | RHOA-homo-535 | CAGCCCUGAUAGUUUAGAATT |
|  |  | UUCUAAACUAUCAGGGCUGTT |
| siRNA 3 | RHOA-homo-360 | CCAGUUCCCAGAGGUGUAUTT |
|  |  | AUACACCUCUGGGAACUGGTT |

Due to the high knockdown efficiency (at both mRNA and protein levels) of siRNA2 and 3, they were selected.
