# Supplementary material for: Mechanism of RhoA regulating benign prostatic hyperplasia: RhoA-ROCK-β-catenin signaling axis and static & dynamic dual roles
Source: Mol Med. 2023 Oct 20;29:139. doi: 10.1186/s10020-023-00734-2 (PMC10589999; doi:10.1186/s10020-023-00734-2)
Supplement: Supplementary file 4 — Additional file 4: Table S4. Primer sequences used for qRT-PCR. [file 10020_2023_734_MOESM4_ESM.docx]

**Additional file 4: Table S4: Primer sequences used for qRT-PCR**

| Gene | Primer | Primer sequence (5’ to 3’) |
| --- | --- | --- |
| RhoA | Forward | AGCCTGTGGAAAGACATGCTT |
|  | Reverse | TCAAACACTGTGGGCACATAC |
| ROCK1 | Forward | AACATGCTGCTGGATAAATCTGG |
|  | Reverse | TGTATCACATCGTACCATGCCT |
| ROCK2 | Forward | TTGCTCTGGATGCAATACACTC |
|  | Reverse | TCTCGCCCATAGAAACCATCA |
| GAPDH | Forward | GGAGCGAGATCCCTCCAAAAT |
|  | Reverse | GGCTGTTGTCATACTTCTCATGG |
