# Supplementary material for: Mechanism of RhoA regulating benign prostatic hyperplasia: RhoA-ROCK-β-catenin signaling axis and static & dynamic dual roles
Source: Mol Med. 2023 Oct 20;29:139. doi: 10.1186/s10020-023-00734-2 (PMC10589999; doi:10.1186/s10020-023-00734-2)
Supplement: Supplementary file 5 — Additional file 5: Figure S1. Immunoblot assay revealed the protein expression level of GTP-RhoA and Total RhoA in normal prostate tissue and BPH tissue. Data were expressed as mean±SD. * p＜0.05. Figure S2. Expression of RhoB and RhoC in human prostate tissue. [file 10020_2023_734_MOESM5_ESM.doc]

**Additional file 1**

**Figure legends**

**Figure S1.** Immunoblot assay revealed the protein expression level of GTP-RhoA and Total RhoA in normal prostate tissue and BPH tissue. Data were expressed as mean±SD. * p＜0.05.

**Figure S2. Expression of** **RhoB and RhoC in human prostate tissue.** (**A**) Immunoblot assay revealed the protein expression level of RhoB and RhoC in normal prostate tissue and BPH tissue. (**B**) Immunohistochemical staining of RhoB and RhoC for normal human prostate and BPH prostate. (**C**) Immunofluorescence staining of RhoB and RhoC for normal human prostate and BPH prostate. DAPI (blue) indicated nuclear staining and Cy3-immunofluorescence (red) indicated RhoB or RhoC protein staining. Data were expressed as mean±SD. * p＜0.05, ** *p*＜0.01, *** *p*＜0.001.
